# Carbon Sequestration of *Caesalpinia platyloba* S. Watt (Leguminosae) (Lott 1985) in the Tropical Deciduous Forest

**DOI:** 10.1371/journal.pone.0125478

**Published:** 2015-05-19

**Authors:** Norma Diaz-Gustavo, Martín Martínez-Salvador, José Luís García-Hernández, Mariano Norzagaray-Campos, Antonio Luna-González, Héctor Abelardo González-Ocampo

**Affiliations:** 1 Instituto Nacional de Investigaciones Forestales (INIFAP), SAGARPA, Campo Experimental Delicias, Km 2, Carretera Delicias-Rosales, Delicias, Chihuahua, Mexico, C.P. 33000, +526394721974; 2 Universidad Juárez del Estado de Durango, Facultad de Agricultura y Zootecnia Km, 35 Carretera Gómez Palacio-Tlahualilo, Venecia, Durango, México, C.P. 35170, +528717118876; 3 Instituto Politécncio Nacional-CIIDIR-SINALOA, Juan de Dios Bátiz Paredes #250, Guasave, Sinaloa, México, C.P. 81000, +526878729626; University of Calgary, CANADA

## Abstract

*Caesalpinia platyloba* was evaluated as an alternative for the retention of atmospheric carbon and as a feasible and viable economic activity in terms of income for tropical deciduous forest (TDF) peasants in the carbon markets. A total of 110 trees of *C*. *platyloba* from plantations and a TDF in the Northwest of Mexico were sampled. Growth (increase in height, diameter, and volume curves) was adjusted to assess their growth. Growth of individuals (height, diameter at breast height [DBH], age, and tree crown cover) was recorded. The Schumacher model (*H* = *β*_0_ *e* ^*β*_1_•*E*^-1^^), by means of the guided curve method, was used to adjust growth models. Information analysis was made through the non-linear procedure with the multivariate secant or false position (DUD) method using the SAS software. Growth and increase models revealed acceptable adjustments (pseudo R^2^>0.8). *C*. *platyloba* reaches >8m of height with 12cm in diameter and 550cm^3^ of volume, presenting the highest increase at 11 years considered as basal age. Highest significant density of wood was in good quality sites (0.80g•cm^-3^), with a carbon content (average of 99.15tC•ha^-1^) at the highest density of 2500 trees•ha^-1^ (without thinning). Average incomes of US$483.33tC•ha^-1^ are expected. The profitability values (NPW = US$81,646.65, IRR = 472%, and B/C = 0.82) for *C*. *platyloba* make its cultivation a viable and profitable activity, considering a management scheme of the income derived from wood selling and from carbon credits.

## Key Message

The paper evaluates and proposes the profitability of *Caesalpinia platyloba* forestry within the carbon credits market as well as the contribution of its cultivation to atmospheric carbon sequestration and to climate

## Introduction

Mexico is considered as a top mega-diverse country [1, 2] due to the large latitudinal and longitudinal expansion of the Nearctic-Neotropical migratory system covering it [3, 4]. Within this large area, ecosystems offer a number of “services” such as biological control of both pests and diseases, pollination of cultivated plants, prevention of soil erosion, hydrogeochemical cycle, and carbon uptake [5–7]. Carbon uptake can be classified in five components: biomass on the soil (trees and understory); biomass beneath the soil (roots); standing dead trees and fallen trunks, and decaying leaves in the soil [8]. In this sense, carbon sequestration has been proposed as a measure to stop or reverse the increase of CO_2_ in the atmosphere [9] or as a strategy to achieve food security worldwide [10].

Arid and semiarid regions are potential carbon sinks [11]. The two most widely suggested options for carbon sequestration are: forestation (planting of trees) and reforestation of grasslands by excluding grazing on them. Specifically afforestation raises the presence of C through a larger and more efficient use of the resources for primary production [12, 13]. This effect is given by increasing the capacity of the area to capture and store carbon [14–16] and increasing biodiversity [17]. In reforestation, by means of planting replacement trees, both exotic [18, 19] and native species [20, 21] are used; however, due to the negative effects that exotic species exert on native ones [22–24], the use of local species is recommended.

Among the native species of the tropical deciduous forest (TDF), *C*. *platyloba* (Palo Colorado, vernacular name) is one of the most prominent tree species of the TDF [25] considered for the reforestation of natural protected areas (NPA) [26]. Palo Colorado pertains to the Fabaceae family. It has ample foliage with non-invasive roots. Foliage covers the tree the whole year and grows fast. Thus, this tree has become a candidate for landscaping [27]. This species is considered as one of the most dense species within the TDF [28]. Besides, this tree has great acceptance in the rustic construction market, particularly for the ecotourism sector, based on its high resistance, durability, and straight shaft [29].

The site index (SI) is defined as the maximal height that trees from a patch of land can reach at a given age and is termed as the base age [30]. This index has been known and used as a practical measure of forestry productivity of a site, being the height of the trees an indicator of volume and potential products of a forestry plantation [31]. The SI has become the most popular and practical method to assess forestry productivity and requires assuming a model that is able to represent the age-height relation of a family of curves generated under the same model [32]. In this way, the base age allows labeling the SI curves, which can be fixed as the profitability point [33] or as the maximum of the average height increase [34]. These parameters can be global, that is, shared by all patches of land, or local, that is, specific for each patch of land. Parameters estimation has been performed with linear or non-linear regression models of fixed effects that assume normality, variance equality and independence of residues. The Schumacher model possesses desirable characteristics to describe adequately the growth patterns in the dominant height observed in forestry masses, using only two parameters: α representing the maximal height (asymptote) and β, which is the rate of growth in height change with age.

In Mexico, *C*. *platyloba* is a species whose ‘‘varas” or stems have been used as plant support stakes in horticultural fields (mainly tomato crops) since the middle of the last century in the Pacific Coast of Mexico [35]. In addition, they have served as fence posts in the Mayan agroforestry for centuries [36] or its bark and leaves have been used as foraging in the “Tierra Caliente” area in southern Mexico [37]. All these properties turn *C*. *platyloba* into a multipurpose tree in the TDF; despite the extensive to intensive agro-forestry use of *C*. *platyloba* species, no approximation to the quantification of biomass carbon stocks has been made. This approximation consists of inferring long-term changes, using regression models to convert inventory data in an estimation of the aboveground biomass (AGB) [38]. In this sense, the economic feasibility for the carbon credits market of an extensive forest plantation of *C*. *platyloba* in the TDF of the Americas depends on its biomass carbon stocks in the long-term and on the generated income. Therefore, we evaluated *C*. *platyloba* as a TDF species, under intensive cultivation conditions, as an alternative for the retention of atmospheric carbon and as a feasible and viable economic activity in terms of income for peasants in the TDF.

## Methods

**Fig 1:**
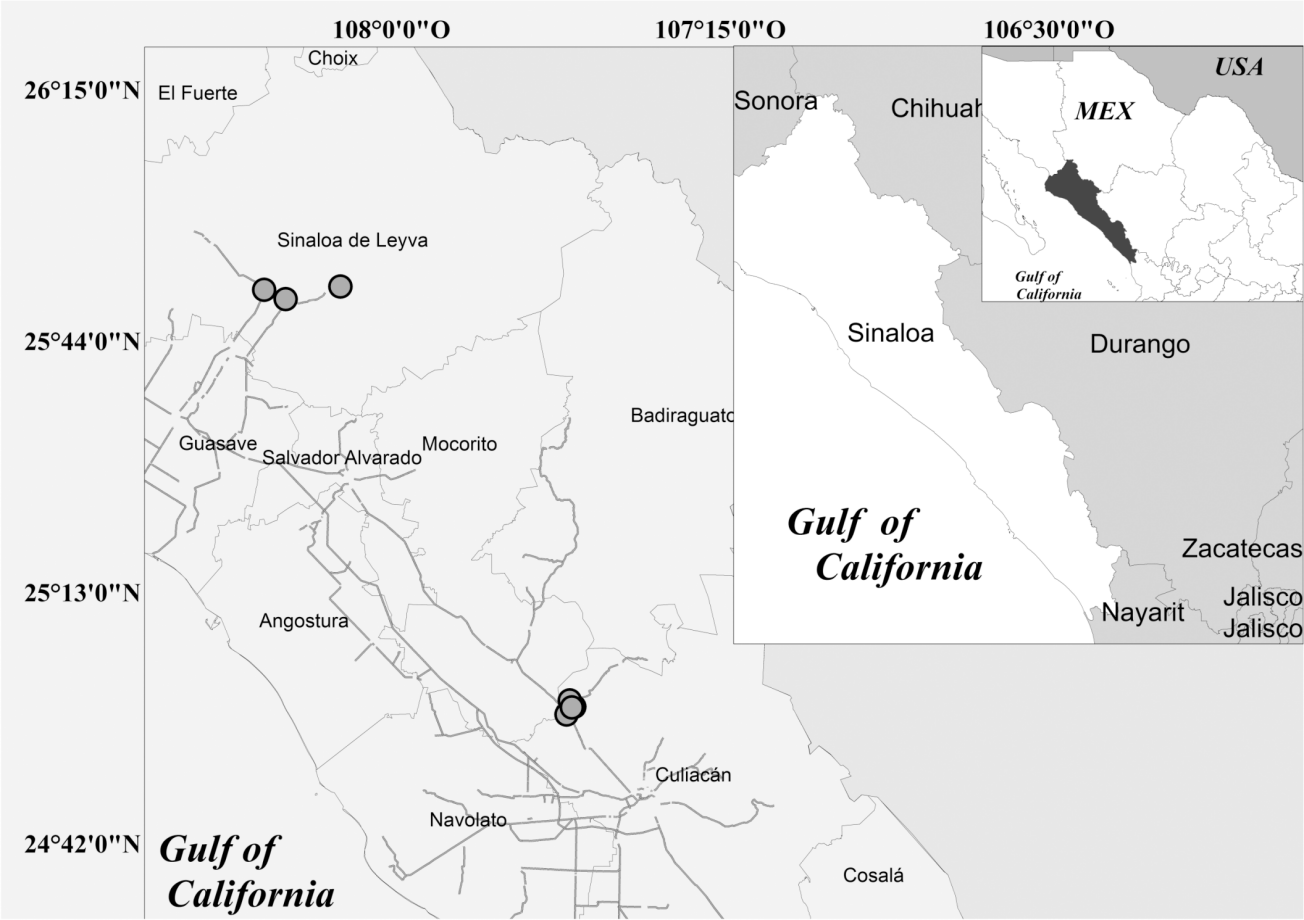
Study area. Collection sites from extensive-forest plantations and from TDF.

During 2011, we measured 110 *C*. *platyloba* trees from plantations and a tropical deciduous forest in Sinaloa, Mexico (Fig 1). Eighty-five trees were recorded in plantations of 1, 2, 3, 5, 7, and 8 years of age (there were no older trees cultivated), choosing dominant or co-dominant trees, that is, defect-free individuals, without damages due to cuts, tree felling, without embryos that could present some type of malformation, dry trunks, bifurcated, suppressed, or other characteristics that could alter their growth. Due to the lack of plantations older than 8 years at the study area, we also measured 25 trees growing in the tropical deciduous forest with similar characteristics of density and structure to the planting areas. Trees of 9, 10, 12, 15, 19, and 21 years of age were measured. The age estimation was made according to records given by the owners of the forest. These trees were chosen following the same attributes of those measured in the plantations areas.

The field studies did not involve endangered or protected species and no specific permissions were required for these locations/activities. The variables measured for each tree were height, DBH (diameter breast height), and the crown cover.

For volumes calculation, each tree was measured standing up by gathering diameters and lengths of straight sections of the stem and also from the branches [39, 40]. The volume of each section was estimated by the use of the Smalian formula instead of the Huber or the Newton formulas. The Newton method is exact for paraboloid, cone and neiloid geometries, whereas the Smalian and the Huber methods are exact for paraboloid, which permits to estimate an average diameter from the extreme diameters of the measured section. In addition, the error differences between the Huber and the Smalian method decrease when *n*→∞ [41], and it allows estimating the volume with lesser errors when the shape of the sections tend to be conic, as in this case. Hence, the volume of each section, including the branches section, was added to calculate the total volume of each tree.

Growth and increment models were adjusted for the construction of growth in height, diameter, and volume curves, as well as for the biomass increase considering the specific weight of the species and taking into account both the aerial parts and the root of each individual. The private plantation, owned by Jesús Ramón Arango Valenzuela, has been established since 2002, and information can be found at: www.conafor.gob.mx:8080/documentos/download.aspx?articulo=1230, +5201667 75056000. We purposefully chose trees of different ages, and sampled trees within the plantation and at the tropical deciduous forest considering the characteristics of dominance and free growth.

### Growth Model

Growth model was adjusted according to the Schumacher model [42], which applies particularly to growth and yield (Eq 1). Estimation of parameters is achieved generally by adopting a logarithmic transformation of the height variable, establishing a linear model whose parameters are provided by ordinary least squares (OLS) [43]. The analyzed trees were distributed within a range of the species’ diametric categories, aimed at obtaining a normal curve. This model possesses desirable features to describe adequately the growth patterns at the dominant height observed in forest masses, using only two parameters: the first represents the maximal height (asymptote) and the second corresponds to the rate of growth change in height with age [44].

#### Current Annual Increment

The volumes of the trees were estimated at different ages to generate a model of growth and volume increase, by means of a growth equation where the increment corresponds to the first derivative of the model. Therefore, the current annual increment (CAI) is the increase in volume at a particular age and is determined by annual measurements of standing volume and is calculated with the following equation:

$$\text{CAI}={\text{Y}}_{\left(\text{t}+1\right)}-{\text{Y}}_{t}$$ (1)

Where: CAI = Current annual increment; Y = Volume at age *t*.

#### Site Index

The site index (SI) is the adjustment of a curve that predicts the growth in height of trees, because growth in height is the most accepted scientific indirect indicator to assess the quality of a site to attain tree growth [45].

To know the different growth rhythms of *C*. *platyloba* in the study area, we adjusted the site index models, which are defined as the dominant height at a reference age (basal age), and is the method most frequently used to evaluate the productivity of forests or to assess their relation with ecological productivity [33].

To adjust the SI curves we employed the guided curve method, used mainly for anamorphic SI curves [46–48]. The obtained data were used to calculate the growth curves in function of expressed biological, edaphological, and climatic characteristics; non-linear regression analysis was performed to model the accumulated growth [42]:

$${\text{H}}_{m}=\beta_{0}{{\text{e}}^{{(\beta }_{1}\cdot E^{-1})}}^{}$$ (2)

Where, H = Height of trees (m); E = Age (years); e = Natural logarithmic base; β_0_ = Asymptotic value parameter, and; β _1_ = Rate of change parameter.

Information analysis was made using the non-linear procedure with the multivariate secant or false position (DUD) method by means of the SAS software. The base age was established at 11 years. At this age, the trees reach about 8 cm of diameter, and then most of them are harvested and used to make fences to support crops such as tomatoes. According to field data and an age base of 11 years, three qualities of site were considered, 8m height for good site index, 6m height for regular, and 4m height for bad sites. From this information, we were also able to know the volume growth of the aerial parts on different quality areas for the studied species.

In order to validate the site index model, we compared the height calculated with the best model versus the height of 10 randomly selected trees with known height-age. Hence, a ratio of variation in the prediction was estimated.

#### Basic Density

Wood basic density or specific gravity (*SG*) is the most important predictor of the mechanical properties, representing the dry biomass per volume unit of live wood [49, 50]. *SG* was calculated with 50 samples of *C*. *platyloba* branches and stems, determining density and constant weights of the wood. Samples cut with machetes had an average length and diameter of 11 and 2cm, respectively. Each sample was kept in a plastic bag. In the environmental analyses, performed at the laboratory of CIIDIR-SINALOA, the fresh weight was determined with a SHIMADZU Model AY220 analytical balance, with a maximal capacity of 0.220 g. Once the samples had been weighed, the dimensional method was applied, measuring the length of each section with a millimeter-graded ruler [51]. Length and diameter values were used to calculate the volume of each sample (Eq 4). Afterwards, samples were introduced in a RIOSSA H5-41 HSML oven on aluminum foil trays, at a temperature of 105 ± 5°C, until reaching the anhydrous weight. Once the *Po* anhydrous weight had been obtained, the green volume or estimated volume, *Vv*, of the wood samples and the basic wood density value, *Db* (g·cm^-3^), were obtained.

$$\text{Vv}=\frac{\pi \cdot {\text{D}}^{2}\cdot \text{L}}{4}$$ (3)

Where: Vv = estimated volume of the wood sample (cm^3^); D = Squared diameter of the wood sample (cm); L = Length of the wood sample (cm).

$$\text{Db}=\frac{\text{Po}\;\left(\text{g}\right)}{\text{Vv}\;\left({\text{cm}}^{3}\right)}$$ (4)

Where: Db = Basic density; Po = Dry weight (g); Vv = Green volume (cm^3^).

#### Aerial Biomass

A dasometric inventory was performed between August and September 2010, recording for each tree the following: basic density, *ρ* (g·cm^-3^) [50]; normal diameter, *DN*_1.30m_ (cm); total height, *H* (m); crown cover, *Cc* (m); diameter of seedlings below 7.5cm; diameter at breast height, *DBH* (trees larger than 7.5cm); age, *E* (years); and volume (m^3^) [52].

Volume estimation of *C*. *platyloba* was calculated for the shafts and, later on, this value was expanded to calculate the volume of other components [53].

$$V=0.7854\times {\text{DAP}}^{2}\times \text{ff}\times \text{L}$$ (5)

Where: V = Volume; DAP = Diameter at breast height; ff = Shape factor; L = Length.

#### Carbon Content

Carbon uptake is based on the growth and increment models adjusted according to the analysis of different age plantations. We determined the carbon content from 50 samples of the wood of *C*. *platyloba* at the Plants, Water and Soil Laboratory (LASPA) of the Graduate College (COLPOS) of the SAGARPA Ministry (Ministry of Agriculture and Live Stock) by means of the dry combustion method (calcination). Once the percentage of carbon content (*%CC*) had been determined, the stored carbon was expressed as *t* in decimal numbers. The biomass value was multiplied by the carbon concentration per tree, yielding the total carbon stored in the aerial parts [54, 55]. The accumulated carbon (captured) per hectare was estimated in function of the volumetric yield through the amount of existing carbon per wood volume [56], taking as reference the following equation [57]:

$$\text{C}={\text{f}}_{\text{c}}\times \text{BWA}$$ (6)

Where: *C* = Carbon stored (in Mg) by the tress; *f*_*c*_ = carbon fraction in the biomass; *BWA* = Aerial biomass (Mg).

Given that carbon credits are expressed in CO_2_ equivalent tons, the equivalence corresponding to the atomic weights of the elements was used. Carbon becomes CO_2_ equivalents (CO_2_e) by multiplying by 44/12 (the relation of the atomic mass of one molecule of CO_2_ to the atomic mass of one carbon atom) [58]. That is, for each ton of absorbed carbon in the forest biomass there is a reduction of 3667 tons of CO_2_ sequestered from the atmosphere [59], this was calculated with the following equation:

$${{CO}_{2}\text{e}}_{}=3.67\times \text{C}$$ (7)

Where: *CO*_*2*_ = equivalents of carbon dioxide; *C* = accumulated carbon (tons); *3*.*67* = constant resulting from the division of the molecular weight of oxygen by the molecular weight of carbon.

#### Estimation of Profitability

The Net Present Value (NPV) is the sum of the present value of all its cash flows, both inflows and outflows, discounted at a rate consistent with the project´s risk [60]:

$$NPV=I_{0}+\frac{I_{1}}{1+r}+\frac{I_{2}}{{\left(1+r\right)}^{2}}+\frac{I_{n}}{{\left(1+r\right)}^{n}}$$ (8)

Where *I* represent the net benefits for each year, "*0*" represents the initial net benefit, "*1*" represents the year one net benefit, and so on. The exponent in the denominator is also equal to each year of the analysis, up to *n*, the number of years in the analysis term. The discount rate is *r* and is held constant throughout the analysis period.

The Internal Rate of Return (IRR) is the discount rate that makes the present value of a net cash flow equal to zero [61]. A project is economically viable when the IRR is greater than the opportunity cost of capital. Pure investment projects have a unique IRR value; otherwise, there are several IRR. IRR is calculated by using the following formula:

$$IRR=\sum_{i=1}^{n}[\frac{\left(B_{i}-C_{i}\right)}{{\left(1+r\right)}^{i}}]=0$$ (9)

Where: *r* is a discount rate, and *B*_*i*_ and *C*_*i*_ are, respectively, the values of receipts and cost at time *i*.

Benefit-cost ratio (B/C) ratio is the comparison of the present value of an investment decision or project with its initial cost. A ratio greater than one indicates that the project is a viable one [62]:

$$\frac{B}{C}=\frac{\sum_{\text{n}=0}^{\text{N}}{\text{B}}_{\text{n}}∕{\left(1+\text{i}\right)}^{\text{n}}}{\sum_{\text{n}=0}^{\text{N}}{\text{C}}_{\text{n}}/{\left(1+\text{i}\right)}^{\text{n}}}$$ (10)

### Statistical Analysis

Statistical analyses were performed using SAS 8.1 for Windows (SAS Institute, Cary, NC, USA). Growth, current annual increment, site index, basic density, aerial biomass, and carbon content of plant materials among age classes were compared by one-way ANOVA, followed by the LSD method to test among different age groups.

## Results

Organic carbon concentrations of foliage and wood of *C*. *platyloba* varied from (43.64±0.79) (mean ± standard error, the same below)% to (53.04±1.00)% and from 44.46±1.00 to 50.66±1.28%, respectively (Table 1). In general, there were no significant differences between foliage and wood (two-way ANOVA, F = 0.532, p = 0.469). Two-way ANOVA showed that species (F = 5.503, p<0.001) and species component organs (F = 5.037, p<0.001) had significant effects on organic carbon concentration. There were no significant differences among decay classes and vegetation types for coarse woody debris (CWD) organic carbon concentrations.

**Table 1:** Parameter of the growth model of *C*. *platyloba* in a TDF of Mexico.

Growth models	TSS	ESS	MSE	F_c_	Pr > F	r^2^	B_0_	B_1_
**Height (m)**	1649.9	1706.1	0.526	1569.3	<.0001	**0.97**	10.14	4.09
**Diameter (m)**	2539.2	2702.3	1.525	832.53	<.0001	**0.94**	12.86	4.25
**Volume (m** ^**3**^ **)**	53840	67682	1293	208.09	<.0001	**0.80**	24.94	17.80

Total sum of squares, TSS; error sum of squares, ESS; mean square error, MSE; calculated F value, F_c_; probability Pr; coefficient of determination, *r* ^*2*^; Estimated parameters through models B_0_ and B_1_.

### Growth Model

The growth model shows how the mean squared error (MSE), the determination coefficient (R^2^), and the probability (Pr) reveal statistically acceptable adjustments (Table 1).

In this way, the growth in height (site index) of *C*. *platyloba* started at a fixed point, followed by a slow phase. According to the validation of the model by comparing the predicted height versus the known age-height of a group of trees, we found a variation of 12±5%.

In *C*. *platyloba*, the increment in mass starts in three quality sites at 3 years and the most important carbon capture occurs at 13 years. The tree starts with an almost nil increase, but at 3 years growth increases almost 10-times in the aerial parts (stem, branches, and leaves) with respect to the first year [63]. The same behavior was observed in growth curves, which revealed that the increase depends directly on the site’s quality, with the highest volume increase at 9 years (Table 1).

**Fig 2:**
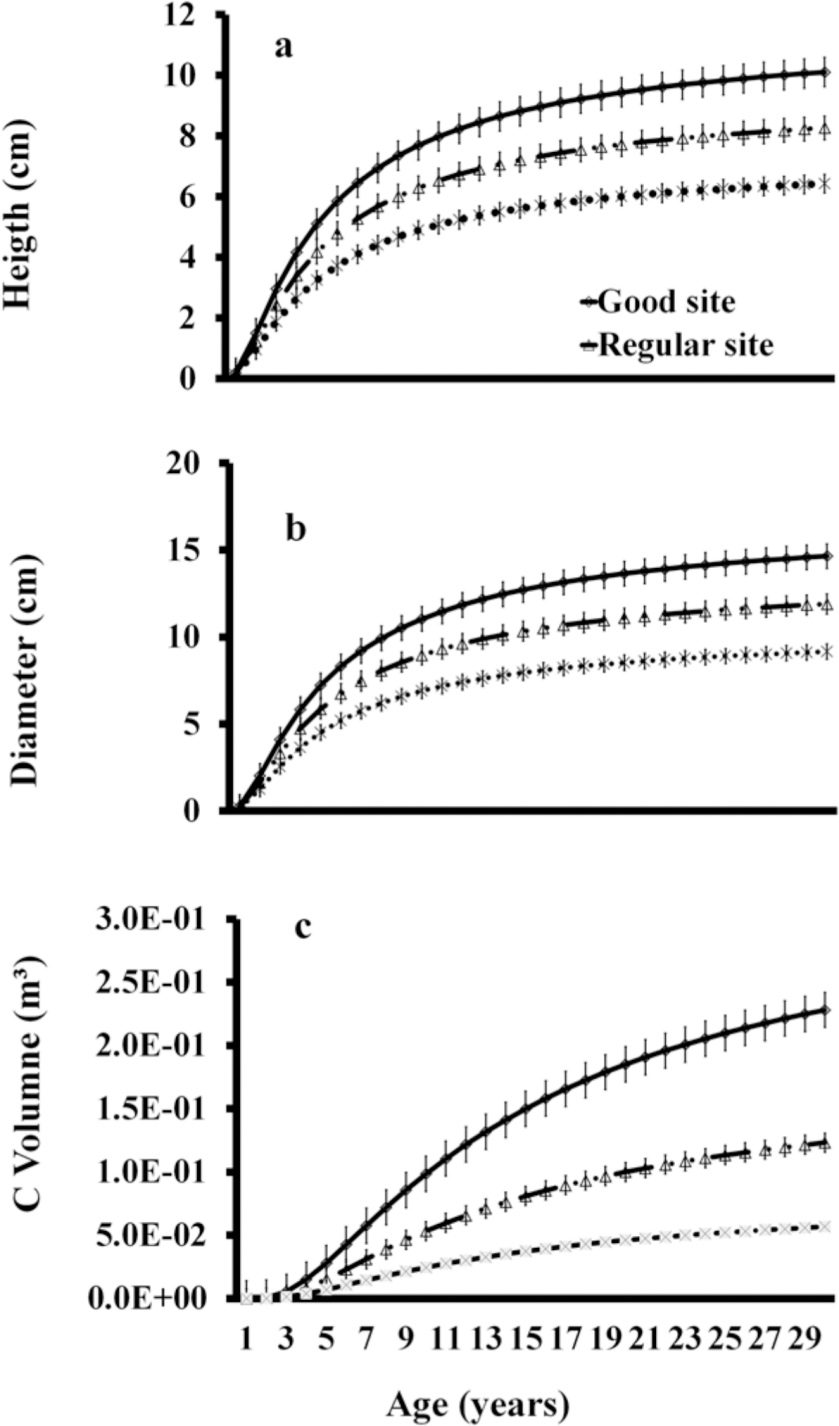
Growth of *C*. *platyloba*, Age-Height (a), Age-Diameter (a), and Age-Volume (a) in good sites (GS), regular sites (RS), and bad sites (BS). Current annual increase, in volume.

*C*. *platyloba* in a 30-year period would reach a height (m) of 10.10±0.48SE, 8.26±0.39SE, and 6.4±0.31SE; a diameter (cm) of 14.6±0.71SE, 1±0.5SE, and 3.3±0.44SE; and a volume (m^3^·tree^-1^) of 1405±0.014SE, 1053±0.01SE, and 702±0.003SE in good, regular, and bad sites index, respectively. One-way ANOVA showed that growth in height (F = 11.17, p<0.05) (Fig 2A), diameter (cm) (F = 13.04, p<0.05) (Fig 2B), and volume (m^3^·tree^-1^) (F = 29.61, p<0.05) (Fig 2C) were significantly higher in good quality sites (Fig 2).

**Fig 3:**
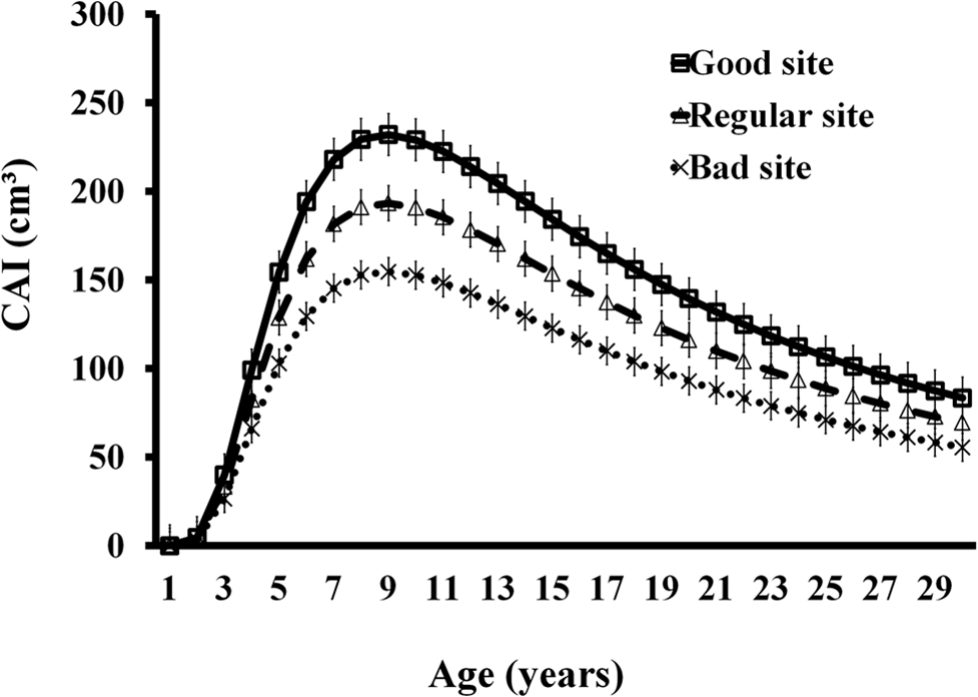
CAI in good sites (GS), regular sites (RS), and bad sites (BS).

With the first derivative of the Schumacher model, the values of current annual increase were obtained. The highest increases in mass occurred in three periods, as follows. At three years, values were 39.98, 33.32, and 26.66cm^3^·tree^-1^; at nine years, they were 232.01, 193.34, 154.67cm^3^·tree^-1^; and at 13 years, they were 204.35, 170.30, and 136.24cm^3^·tree^-1^, in sites with good, regular, and bad qualities, respectively. In the three types of sites, the CAI in volume started to decrease at 13 years. One-way ANOVA showed that CAI (F = 5.64, p<0.05) were significantly higher in good quality sites (Fig 3).

### Basic Density, *Db* (cm^3^·tree^-1^) of *C*. *platyloba* Wood

From the average values obtained from the 50 wood samples for basic density determination from the dry combustion method (calcination), a green volume of 40.82cm^3^ and an average anhydrous weight of 25.54g were estimated. Average basic density was of 1.24, 0.76, 0.65, 0.65, and 0.68cm^3^ tree^-1^ for 20, 12, 7, 5, and 3 years, respectively, with an overall average basic density of 0.80g·cm^-3^·tree^-1^.

### Aerial Volume (*m* ^*3*^·tree^-1^)

**Fig 4:**
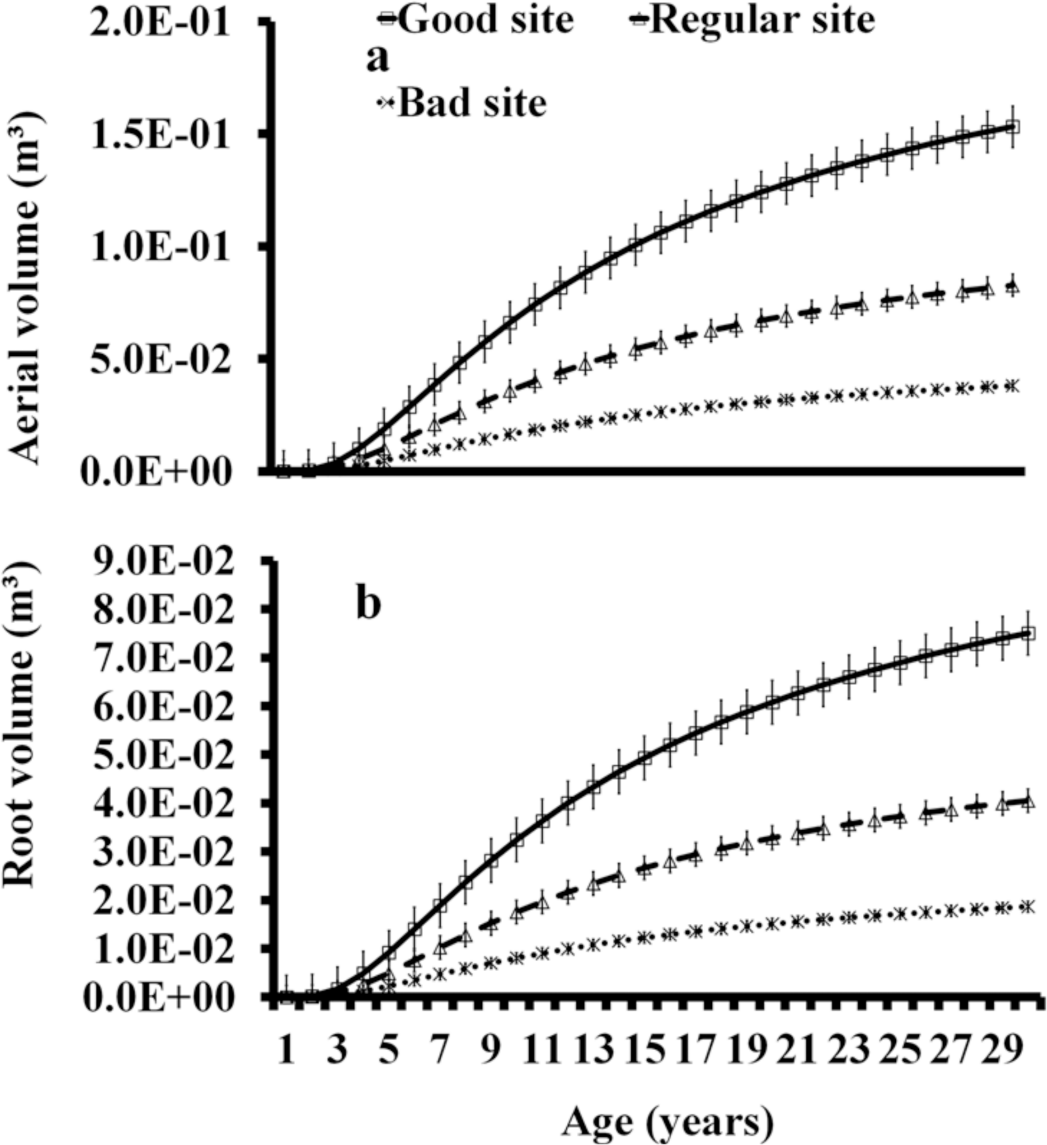
Estimations of total aerial and root volume (m^3^·tree-1) of *C*. *platyloba* at 10 years. The figure depicts the behavior of volume according to the site’s quality.

The aerial volume was determined for each individual from 1 to 30 years, considering a 0.70 value for the taper factor [57] and biomass expansion factor of 0.87 [64]. Volume calculations [53] revealed that it increased with age. In the three quality sites, this increase was slow during the first 3 years; it accelerated from the 4th to the 13th year, from where it slowed down again until 30 years. One-way ANOVA showed that aerial volume (m^3^·tree^-1^) (F = 29.61, p<0.05) was significantly higher in good quality sites (Fig 4A).

### Root Volume

An *fc* of 40% was calculated according to Rincon et al. [65]. In woody systems, such as forests, the root crown comprises in average 41% of total root biomass [66]. In addition, ANOVA showed no significant differences between the two parts of the tree, the root volume was 0.059m^3^·tree^-1^ in good quality sites at the base age of 10 years. Average root volumes (m^3^·tree^-1^) of 0.0442±0.0045SE, 0.023857081±0.0024SE, and 0.011±0.0011SE were obtained for GS, RS, and BS, respectively. One-way ANOVA showed that root volume (m^3^·tree^-1^) (F = 29.60, p<0.05) was significantly higher in good quality sites (Fig 4B).

### Total Volume (m^3^C·tree^-1^)

**Fig 5:**
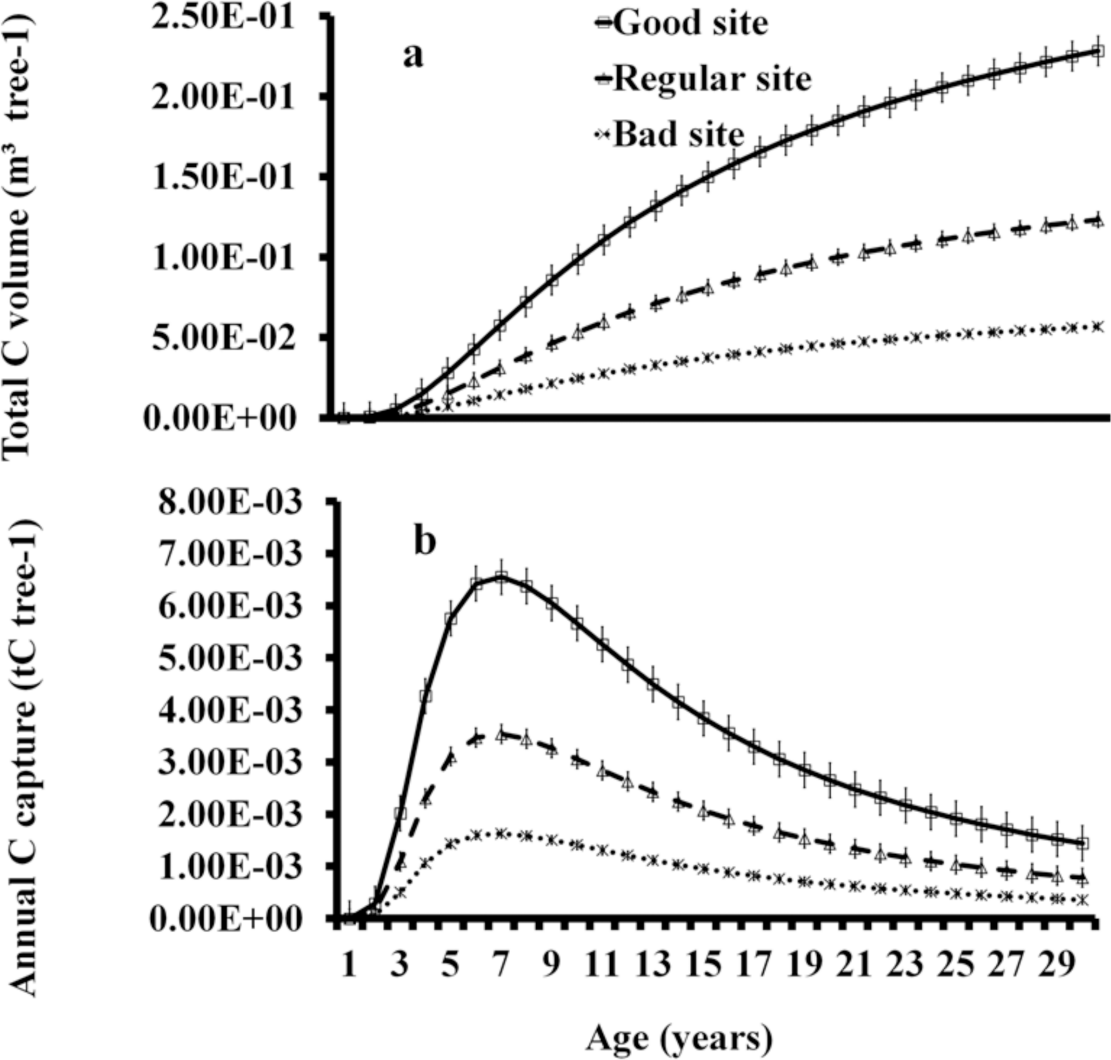
Estimation of total carbon sequestration (a) and stored carbon (b) for *C*. *platyloba* in a 30-year period in good, regular, and bad quality sites.

Average volume *per* tree (m^3^tree^-1^) was 0.134±0.014SE, 0.073±0.007SE, and 0.033±0.003m^3^·tree^-1^ (mean±standard deviation). Average aboveground carbon capture (AGB) per tree was 0.148, 0.08, and 0.037±0.056m^3^·tree^-1^. Average root carbon capture (BGB) per tree was 0.059, 0.032, and 0.015m^3^·tree^-1^. Total volume (aerial parts and root) was estimated in m^3^·tree^-1^ for *C*. *platyloba* at 10 years. Considering the quality of the sites, total biomass of 0.207, 0.112, and 0.051 was obtained for good, regular, and bad sites, respectively. One-way ANOVA showed that carbon volume concentration between aboveground and root (F = 3.28, p = 0.109) had no significant effects on organic carbon concentration. In the same sense, one-way ANOVA showed that carbon volume concentration among quality sites (independently from the tree) (F = 1.80, p = 0.0.24) had no significant effects on organic carbon concentration (Fig 5A).

### Annual Carbon Capture *per* Tree (tC·tree^-1^)

Annual average carbon content was of 0.003, 0.002, and 0.001tC·tree^-1^ for GS, RS, and BS, respectively. For 3, 5, 7, and 12 years, values were 0.96, 0.973, 0.963, and 0.974tC·tree^-1^, respectively. Estimation of carbon uptake by *C*. *platyloba* was obtained in function of the total volume and of a 55% carbon content, calculating the carbon content present in the aerial parts and root, and the total estimation expressed in tC·tree^-1^ [57]. The carbon content stored in the aerial parts (foliage and wood) at 10 years was of 0.075, 0.041, and 0.019tC·tree^-1^ in good, regular, and bad quality sites, respectively. Regarding root biomass, the values were 0.030, 0.016, and 0.007tC·tree^-1^ good, regular, and bad quality sites, respectively.

Organic carbon analysis based on the dry weight revealed an average value of 0.970 of carbon content. For 3, 5, 7 12, and 20 years, the values were 0.96, 0.973, 0.963, 0.974, and 0.971, respectively. The carbon content stored in the aerial parts at the base age (11 years) was of 0.075, 0.041, and 0.019tC·tree^-1^ in good, regular, and bad quality sites, respectively. Regarding root biomass, the carbon uptake was of 0.030, 0.016, and 0.007tC·tree^-1^ in good, regular, and bad quality sites, respectively. Estimation of accumulated carbon in the root was obtained by adding the aerial parts; thereby obtaining the total values that *C*. *platyloba* accumulates in the whole plant in tons of carbon per tree. The minimal value of captured carbon by the root biomass during the first 10 years was of 0.026 and the highest value was of 0.549tC·tree^-1^ at 30 years (Fig 5B).

The total accumulated biomass at densities of 2500, 1100, and 750 trees·ha^-1^ until 10 years of forestry management reveals that, at good, regular, and bad quality sites, the stored carbon was of 263.67, 142.42, and 65.54 tC·ha^-1^, respectively. At all ages, the highest uptake values were found in good quality sites, whereas the lowest values were generated in bad quality sites (Fig 5B). One-way ANOVA showed that annual carbon capture per tree (tC·tree^-1^) (F = 29.61, p<0.05) and total accumulated biomass (F = 30.5, p<0.05) were significant higher in good sites.

### Estimation of Carbon Credits

The price for carbon sequestration of US$5.00 $5·tCO_2_e was taken as reference price, as managed by the World Bank Group [67]. In this way, to estimate the carbon credits we considered the carbon stocks previously presented. Estimation of carbon credits (in US currency) reveals that, at 10 and 30 years, incomes can reach US$1318.37 and US$222.73 per ha, respectively.

According to the cash flow from revenue and costs of the planting project, the calculation of net present value (*NPV*), with a discount rate of 8%, yielded *NPV* = US$5831.9. The *IRR* registered a value of 472%, so this rate of return exceeds the value of the discount rate used in this project, and taking into account the costs of planting and revenues of the project to this date and considering the discount rate of 8%, a *B/C* = 4.82 was obtained.

## Discussion

Growth of *C*. *platyloba* until the age of 7 years occurs at an average rate of 0.34±0.29m per year, diminishing to a rate much lower than 0.09m per year until reaching 20 years, which is similar to other arid zone species [18, 68]. Comparison of *C*. *platyloba* growth with other species of the same climatic conditions reveals differences. On one side, its growth was similar to that of *Astronium graveolens*, *Dalbergia retusa*, *Swietenia macrophylla* [68], and *Acacia salicina* [18]. However, when compared with species of the same genus, such as *C*. *eriostachys* and *C*. *velutina*, its growth was lower at 6 years, since those species reach 13.6 and 9.28m, respectively, at that age [55]. Notwithstanding, growth of *C*. *platyloba* can be higher to that of other species of arid zones, such as *Flourensia thurifera*, *Gutierrezia resinosa*, *Heliotropium stenophyllum*, and *Acacia saligna*, which do not exceed 3m [13].

At 12 years, its size was smaller than that of other species used in forest plantations, such as *Calophyllum brasiliense*, *Vochysia guatemalensis*, *Hyeronima alchorneoides* [69], and *Tectona grandis* [46, 70], and other species such as *Morus alba*, *Leucaena leucocephala*, *Dalbergia sissoo*, *Gliricidia maculata*, *Michelia champaca*, *Samania saman*, and *Albizzia procera* [70]. However, these species are from lowland tropical forests or from subhumid tropical climates and can reach from 15 to 50m in height at this age. Even so, *C*. *platyloba* in comparison to species from subhumid tropical climates, like *Azadirachta indica*, can reach similar o even higher heights [70].

At 20 years, *C*. *platyloba* shows higher height values with respect to values recorded for other timber species of the same genus like *C*. *alata*, *C*. *dulce*, and *C*. *eriostachys* [55]. Other forest species, such as *Tectona grandis*, present at this age heights above 15m [48]. The higher or lower values in size depend, as has been said, on the site’s quality. Semiarid zones, as the one where the studied species strives, maintain environmental conditions where carbon flow is lower [71], turning them into sites of lower quality as compared to humid or temperate tropical regions [38, 72, 73].

Although it cannot be assumed that forest biomass increases in correlation to the density of wood of the constituting species or that the stored carbon will diminish as a result of the decrease in wood density in a given ecosystem [73], the relation between growth and carbon capture in *C*. *platyloba* is favored during its first years of life. During this period, the quality of the ecosystem has been related to the carbon storing capacity [74], obtaining significant adjustments and high determination coefficients in good quality sites. According to the Schumacher model, *C*. *platyloba* is a fast growing species [65], although other studies consider it of slow growth [64]. Variation of this semiarid species is given mainly by the environmental conditions under which the research was undertaken, i.e., the quality of site influences the growth and carbon storing capacity [65] and the soil type. In this study, the aboveground organic carbon concentration (71%) in *C*. *platyloba* was higher in vertisols than in karst soils (between 56% to 65%) [75], but lower than in sandy coastal soils (between 65%-85%) [76] or in leptosols, luvisols, and regosols [77], at similar ages.

The density value obtained for *C*. *platyloba* coincides with reports for trees in natural areas with 0.092g·cm^-3^ [65]. The obtained 0.080g·cm^-3^ value is influenced by the fact of being a forest plantation and by the studied ages; density of wood depends on the quality of the site where they are planted and is directly proportional to the age of the assessed individuals.

The carbon content of *C*. *platyloba* was 0.51% above that reported by Hernández and Torres (2003). Carbon estimations for *C*. *platyloba* at 10 years corresponded to the species under the environmental conditions of the sites where evaluations were made. This is similar to variations in carbon storage values for species of cloud forests [78], secondary forest [79], and forest plantations [80, 81]. This means, as Sandra et al. [82] estimated in tropical trees in Mexico, that carbon storage in *C*. *platyloba* depends on the density per hectare, age of the plant, quality of the site, diversity of species, and management of the plantation or of the forest. In addition, the aboveground stem carbon densities (188.32, 101.75 and 46.814 tC·ha^-1^ at 10 years in good, regular, and bad sites, respectively) were similar to those reported for other TDF trees in India (87–151 tC·ha^-1^) and this carbon increment varies widely among the species and sites [83].

Finally, the income calculated from carbon credits in *C*. *platyloba* plantations covers a large part of the costs generated during the planting period until 15 years, although it is more profitable when no intensive management costs are incurred as in agricultural crops. Notwithstanding, when Téllez *et al*. (2008) evaluated an *Eucalyptus* plantation at a carbon price of US$2.50 tC·ha^-1^ in a 15-year period, the benefits of the net present value and the cost/benefit relation increased, without improving the profitability of the plantation, resulting non-attractive for the owners. That is, profitability of a forest plantation, independently from the cultivated species, will always depend on the value of the ton of carbon in the markets and its price should be well above US$2.50 tCO_2_e·ha^-1^, in order to be attractive to the investors.

## Conclusions

The Schumacher model, by means of the guided curve method, yielded the adjustments to fulfill the assumed regression values, revealing a correct prediction of *C*. *platyloba’s* growth. The curves generated with the model of the site index will allow for a better management of the plantation and for the identification of the most adequate profitability level at the first seven years, since younger plantations have large potential for enhancing terrestrial ecosystem carbon sequestration [84]. In addition, based on the aboveground biomass contribution of *C*. *platyloba* (71%), it can be placed among the top 10 species for Subtropical Evergreen Broad-Leaved Forest described by Lin et al. [85]. The performed dasometric inventory will allow a precise estimation between the growth data and the classification according to site index, whereas data on the common annual increase for *C*. *platyloba* would help to provide feedback to the management programs and silvicultural treatments of this species. Nevertheless, it is recommended to perform a biorational handling of the species in intensive plantations of 2500 trees·ha^-1^ at the beginning. Thereby, before competitions arise among them, at 5 years with a height of 3m and a diameter of 4cm, it is recommended to exploit 1250 trees for the horticultural demand to be used as fence posts, generating an additional income, doing this at intervals according to the demand of wood. Finally, the carbon density in *C*. *platyloba* at 10 years (2500 trees·ha^-1^ with a 263.67 tC·ha^-1^) can yield revenues higher than US$1318.35·ha^-1^, this amount can be considered as a "business as usual" system [86], on sites with good quality and without expenses in fertilizers, irrigation, fuels, and lubricants.
